# A comparison of machine learning methods to classify radioactive elements using prompt-gamma-ray neutron activation data

**DOI:** 10.1038/s41598-023-36832-8

**Published:** 2023-06-19

**Authors:** Jino Mathew, Rohit Kshirsagar, Dzariff Z. Abidin, James Griffin, Stratis Kanarachos, Jithin James, Miltiadis Alamaniotis, Michael E. Fitzpatrick

**Affiliations:** 1 Faculty of Engineering, Environment and Computing, Coventry University, Priory Street, Coventry, CV1 5FB UK; 2 Factory of the Future Advanced Manufacturing Park, University of Sheffield AMRC, Wallis Way, Catcliffe, Rotherham, S60 5TZ UK; 3 Nissan Technical Centre, Cranfield Technology Park, Moulsoe Road, Cranfield, Wharley End, Bedford, MK43 0DB UK; 4 Applied Artificial Intelligence Laboratory, Department of Electrical and Computer Engineering, University of Texas at San Antonio, San Antonio, TX USA

**Keywords:** Characterization and analytical techniques, Mathematics and computing

## Abstract

The detection of illicit radiological materials is critical to establishing a robust second line of defence in nuclear security. Neutron-capture prompt-gamma activation analysis (PGAA) can be used to detect multiple radioactive materials across the entire Periodic Table. However, long detection times and a high rate of false positives pose a significant hindrance in the deployment of PGAA-based systems to identify the presence of illicit substances in nuclear forensics. In the present work, six different machine-learning algorithms were developed to classify radioactive elements based on the PGAA energy spectra. The model performance was evaluated using standard classification metrics and trend curves with an emphasis on comparing the effectiveness of algorithms that are best suited for classifying imbalanced datasets. We analyse the classification performance based on Precision, Recall, F1-score, Specificity, Confusion matrix, ROC-AUC curves, and Geometric Mean Score (GMS) measures. The tree-based algorithms (Decision Trees, Random Forest and AdaBoost) have consistently outperformed Support Vector Machine and K-Nearest Neighbours. Based on the results presented, AdaBoost is the preferred classifier to analyse data containing PGAA spectral information due to the high recall and minimal false negatives reported in the minority class.

## Introduction

There are growing concerns over the threat from nuclear terrorism caused by illicit trafficking and other malicious acts involving nuclear and radiological materials, especially those originating from special nuclear material (SNM). Nuclear security must adapt and improve to combat these threats by using a multi-layered defence model that includes robust prevention, detection, and response mechanisms^1^. The detection of illicit materials is crucial to safeguard a robust second-line of defence that involves screening of potential threats at borders, ports, airports, and nuclear facilities. Henceforth, development and deployment of nuclear security systems with proven detection technologies will remain a key priority^2^. Nuclear-forensics-based analytical methods are increasingly being used to investigate radiological material for its isotopic and elemental composition that lead to useful inferences over the origin of the material. Gamma and neutron detection instruments are predominantly used for identifying the presence of illicit material due to their greater penetration capabilities compared to other types of radiation. However, reducing the false alarm rate is one of the major challenges with such detector systems.

**Figure 1:**
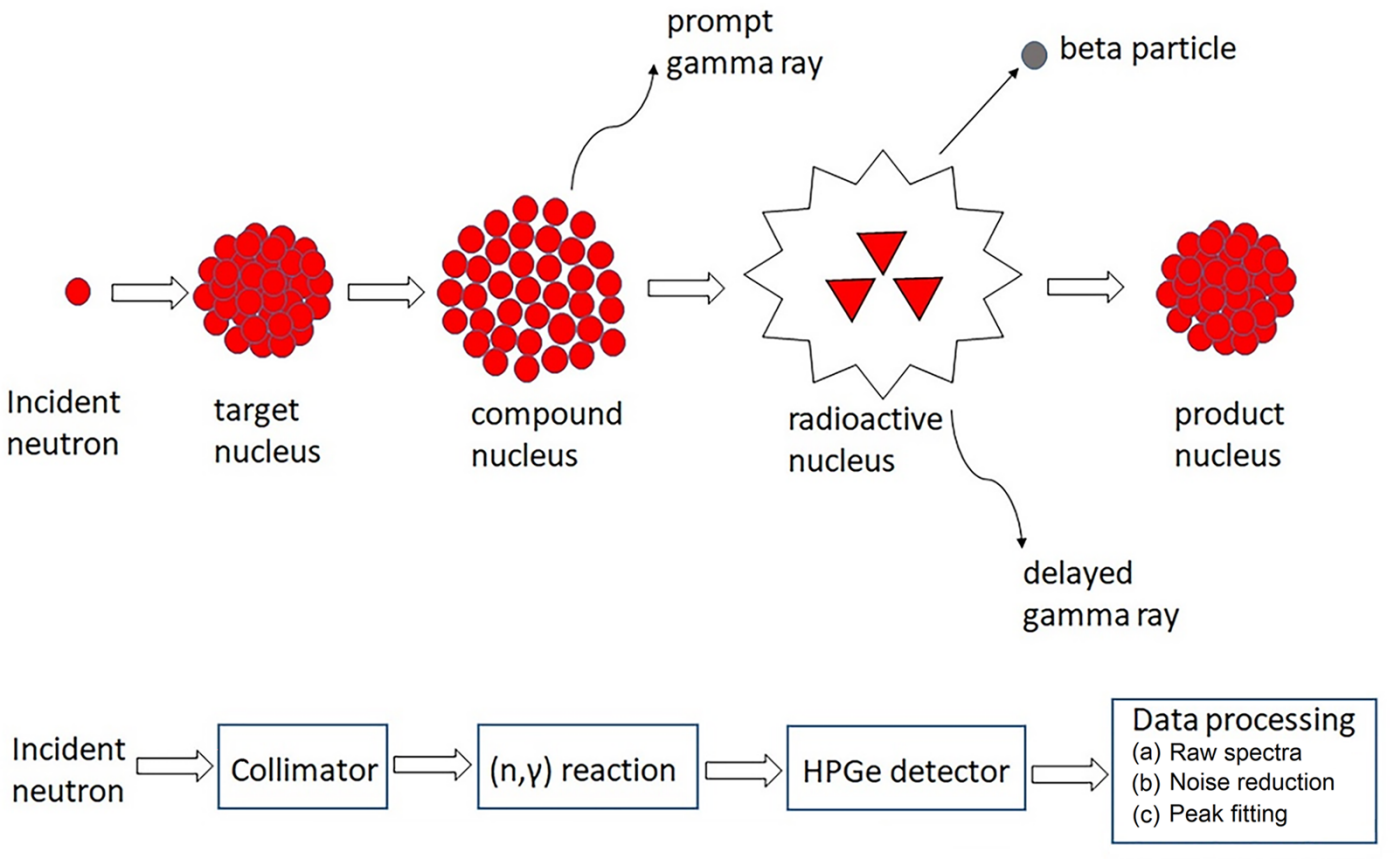
Schematic illustration of prompt-gamma-ray neutron activation analysis (PGAA).

Prompt-Gamma Activation Analysis (PGAA)^3–5^ is an efficient non-destructive radio-analytical technique used for detecting elements via excitation of the target nuclei resulting in the generation of prompt-gamma-rays from neutron capture during the gamma emission (n, *γ*) reaction. PGAA can perform rapid in situ multi-elemental detection of radioactive materials, using *γ*-ray detectors typically made with high-purity germanium (HPGe)^6^. The gamma-ray spectra are subsequently processed and analysed using an electronic data acquisition system. Further, the composite elements of the target samples can be identified by comparing the energies and peak intensities with the PGAA library of prompt-gamma-rays emitted by individual elements. Figure 1 shows a schematic representation of the PGAA technique and presents an illustration of various stages of the elemental detection. PGAA exploits the prompt capture gamma rays directly by triggering a radioactive capture reaction between a neutron and the nucleus of any atom. The neutron then interacts with the target nucleus and a compound nucleus is formed in an excited state. PGAA exhibit relatively complicated gamma spectra and there is interference from the neutron source and background radiation. In pulsed neutron sources, the detection limit of PGAA relies on the effective capture cross-section through (n, *γ*) reaction of the target sample. The major drawback of PGAA is that the high *γ*-ray background from the epithermal neutron beamline can cause prolonged detection times in order to obtain acceptable elemental peaks for low signal amplitudes^7^. The detection time can be significantly reduced by enhancing the signal-to-noise ratio through noise reduction techniques to yield reliable results in real time. Furthermore, the calibration process involving the comparison of the observed spectra in the investigated material with a standard sample spectrum can be quite cumbersome^8^. Moreover, the rate of false positives for detection of any illicit material remains quite high in PGAA^6^.

Machine learning has been extensively used to analyse gamma-ray spectrometry data for identification of radioisotopes and detection of explosives. Alamaniotis et al. developed a fuzzy logic radioisotope identifier^9^ and a hybrid fuzzy genetic approach for rapid identification of spectral peaks in complex gamma ray spectra^10^. Fatah et al.^11^ proposed a Levenberg-Marquadt non-linear least-square fitting for analysis of gamma ray spectra. Varley et al.^12^ used a combination of principal component analysis and neural networks for mapping activity and spatial distribution of ^226^Ra. Sullivan et al.^13^ and Kamuda et al.^14,15^ presented an automated isotope algorithm in a large dataset of low-resolution gamma ray spectra containing a mixture of multiple radio-isotopes based on Bayesian and artificial neural networks respectively. Specifically, PGAA data have been analysed via applying machine-learning methods for detection of illicit materials^16–18^, online monitoring^19^, material classification^20^ and thermal neutron beam optimisation^21^. Recently, Kamuda et al*.* ^22^ compared the performance of artificial neural networks and convolutional neural networks for automated gamma-ray spectroscopy to identify mixtures of radioisotopes. However, there is no comprehensive study to-date comparing the performance of different machine learning methods for classifying PGAA spectral information. Such studies^23–25^ can throw light on the efficacy of different machine learning algorithms that can be potentially beneficial for reducing false alarm rates within detector systems. Additionally, multi-class classification of datasets with skewed distributions represents a major challenge in machine learning^26^. The use of sampling techniques and extended metrics can be effectively used to minimise the discrepancies arising from imbalanced classes^27–29^. However, over-sampling can further increase the computational burden and under-sampling can potentially result in loss of valuable information. Recently, Nega et al.^30^ reviewed different methods primarily based on resampling and classifier adaptation for handling imbalance in multi-label classification whilst addressing its advantages and limitations. An important outcome of this study was developing a comprehensive framework for handling class imbalance is still under-studied despite the increasing application of multi-label classification in different domains.

Elemental identification in PGAA is based on comparing the sample spectrum with the gamma ray database consisting of majority of elements from hydrogen to uranium. The elements are then qualitatively identified based on the energies of the dominant peaks. Large number of gamma rays may be required to redundantly identify an element. Furthermore, to ensure reliability of the element identification, appropriate statistical comparison of the energies, comparison of the relative gamma-ray intensities in the database, and consideration of possible background contaminants from the (n, γ) reactions in the surrounding material have to be undertaken. In addition, the background spectra are used to correct the analysis when necessary. Overall, this can be a tedious and time-consuming process, hence we used ML algorithms to effectively classify the radioactive elements by identifying the underlying patterns in the PGAA spectral data and constitute the rational of this study. In this work, six different machine learning algorithms were developed to undertake classification of radioactive elements with imbalanced classes using PGAA spectral data. Radioactive elements positioned at different locations of the periodic table that are more likely to be stolen and used in illicit activities such as cobalt (_27_Co), caesium (_55_Cs), iridium (_77_Ir), uranium (_92_U) and thorium (_90_^Th^) were classified based on the PGAA energy spectra (*E*_ɣ_) and capture cross-section (*σ*). The most common classification algorithms in supervised learning that are also diverse in nature, For instance, K-Nearest Neighbours are based on nearest neighbours, Artificial neural networks (Deep learning), Decision trees (Tree based learning), AdaBoost (Tree based gradient boosting), Random Forest (Bootstrap aggregation of Trees) and Support vector machines (Kernel based learning) were used to perform robust comparison studies with a focus on evaluation metrics. We evaluate the model performance based on conventionally used classification metrics and trend curves. We also use extended metrics to evaluate the effectiveness and to enhance the understanding of different machine learning algorithms that are best suited for classifying PGAA spectral information.

In the following sections, we discuss the PGAA data in detail, followed by the different supervised algorithms and cross-validation techniques used in this work. In the subsequent sections, we present the results and discussion of the study covering various classification metrics and trend curves used for critical comparison of the applied machine learning algorithms.

## Methods

### PGAA database

The PGAA database^3^ was developed for elemental analysis by the coordinated efforts of the International Atomic Energy Agency (IAEA) and the International Nuclear Data Committee (INDC) are representative of scenarios found in nuclear security and safeguards. The PGAA database (data can be accessed using the supplementary file provided) comprises a wide range of neutron source facilities that can be broadly divided into neutrons from nuclear reactors, and smaller mobile systems such as neutron generators, accelerators, and isotopic sources. The largest source of radiative neutron capture gamma-ray cross sections is the Institute of Isotope and Surface Chemistry, using the thermal-neutron beam facility of the Budapest Research Reactor^31^. The different radioisotopes generated during neutron activation are categorized by different γ–ray energies and half-lives. The neutron energy spectrum and fraction of epithermal neutrons have a strong influence on the measured capture rate. It is widely understood that the specific element that captures a neutron will decay promptly by emitting a γ-ray of energy *E*_γ_. Further, radioisotopes can be analysed quantitatively based on their peak intensities associated with a specific γ-ray energy. The partial capture cross section of the nucleus (*σ*_γ_ (*E*_γ_)), is expressed as the product form P(*E*_γ_).*σ*_0_; where P(*E*_γ_) is the absolute γ emission probability (γs emitted per capture) of the prompt gamma ray of energy *E*_γ_ and *σ*_0_ is the 2200 ms^–1^ neutron capture cross-section.

The elemental capture cross-section in a partial form for an element *Z* is expressed as:

1 $$\sigma_{\gamma }^{Z} \left( {{\text{E}}\gamma } \right) = \theta {\text{P}}\left( {{\text{E}}\gamma } \right)\sigma_{0}$$

where *θ* is the isotopic abundance. Note, the term $\sigma_{\gamma }^{Z} ({\text{E}}_{\gamma } )$ refers to the cross-section required to generate a specific gamma-ray of energy *E*_γ_. The effective capture cross-section can be then defined as the averaged cross-section over the neutron energy spectrum using:

2 $$\mathop {\mathop \sigma \limits^{ \wedge } = \frac{1}{\nu 0}}\limits^{{}} \frac{{\int_{0}^{\infty } {n(v)\sigma \gamma (v)vdv} }}{{\int_{0}^{\infty } {n(v)dv} }} = \frac{1}{v0}\int_{0}^{\infty } {\rho (v)\sigma \gamma (v)vdv}$$

where *v* is the neutron speed and *v*_0_ equals 2200 ms^–1^, *n*(*v*)*dv* is the number density ofneutrons with speed between *v* and *v* + *dv*, *σ*_*γ*_(*v*) is the neutron capture cross-section that is dependent on the speed of the investigated nuclide, and *ρ*(*v*) is the neutron speed distribution function after normalization. In this study, the energy of the γ–ray spectra (*E*_γ_) and effective capture cross-section ($\mathop \sigma \limits^{ \wedge }$) data are used to develop the classification models.

The features were standardised by removing the mean and scaling to unit variance.

The standard score of a sample x is calculated as:

3 $${\text{z}} = \left( {{\text{x }} - \, \mu } \right)/{\text{s}}$$

where µ is the mean of the training samples, and s is the standard deviation of the training samples.

**Figure 2:**
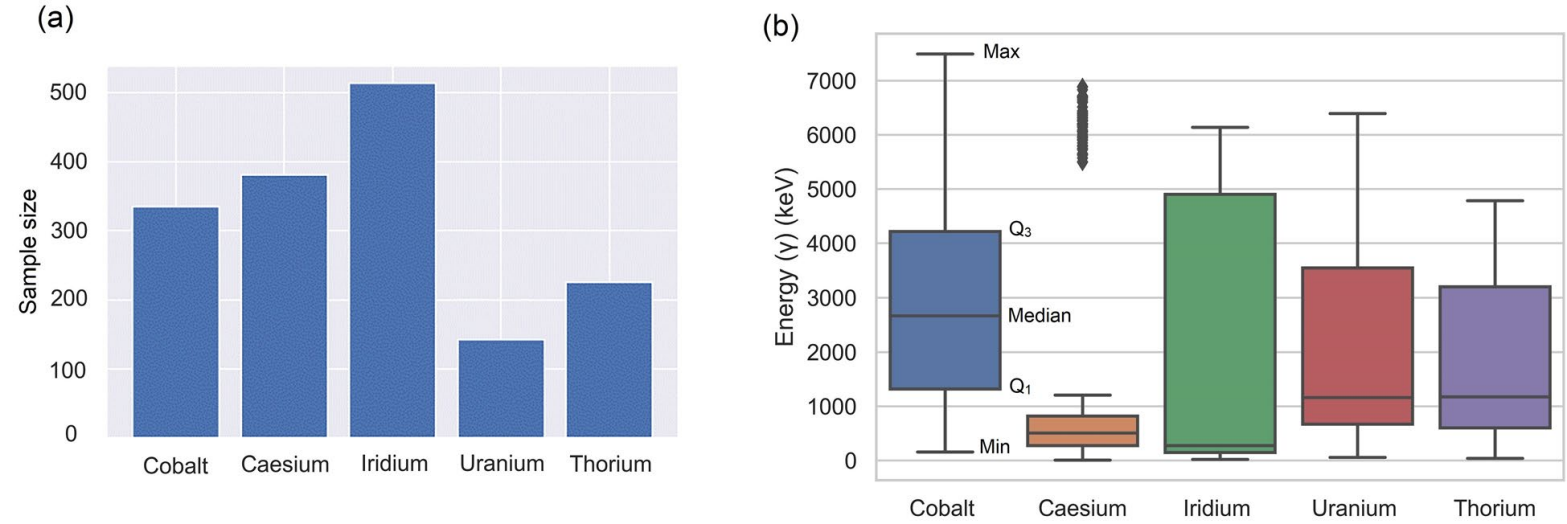
(**a**) Data visualisation of PGAA showing different classes. The proportion of minority class in the dataset was less than 25% of the majority class signifying the presence of moderate class imbalance (**b**) Box plot showing the distribution of Energy spectra for different radiological elements analysed in this study. Box plot represents the five number summary of the data showing the Min, Q1 (lower quartile), Median, Q3 (upper quartile) and Max value.

Figure 2 shows a graphical representation of PGAA data using bar charts showing sample size of different classes (1600 samples were used for training and testing the algorithms with a percentage split of 70:30). The proportion of minority class in the dataset was found to be less than 25% of the majority class, signifying the presence of moderate class imbalance. The majority classes can thereby have a detrimental effect on the learner’s behaviour and can adversely affect the classifier’s performance in the minority classes^32^. In the present paper, we focus on comparing the classifier performance while also identifying the most effective algorithms and metrics when dealing with imbalanced datasets.

### Machine learning algorithms and optimisation strategy

Multi-class classification of imbalanced data represents a major significant challenge, especially in predicting minority class examples due to the skewed distribution of output classes. Suitable optimisation strategies must be undertaken to prevent overfitting and to address the bias-variance trade-off. This involves tuning the hyper-parameters of the machine learning algorithms and implementing an appropriate cross-validation mechanism^33^. In the present work, machine learning algorithms are developed in the Python scripting language using the Scikit-learn library^34^ with the default parameter settings. The hyper-parameters of individual machine learning models were optimised using the *GridSearchCV* routine. Table 1 shows the parameter details of different classification algorithms used in this work. Machine learning algorithms^35^ work on the fundamental principle of learning from representative examples to correlate different features through optimisation of an objective function. K-Nearest Neighbours (KNN) is possibly the simplest algorithm presented in this study, that works based on the idea of proximity (‘Minkowski distance’). Artificial Neural Networks (ANNs) learn by abstract representations of mapping features to targets using activation functions and feedback signals from successive layers. Support Vector Machine (SVM) works based on finding a hyperplane in a given feature space that can best separate the different classes. With SVM, we used the one-vs-rest (ovr) method for multi-class classification where a multi-class classification problem is split into multiple binary classification problems. By contrast, tree-based models such as Decision Tree (DT), Random Forest (RF) and AdaBoost (AB) are a class of non-parameterised models that work by generating a set of splitting rules to partition the feature space into a number of non-overlapping regions. Random Forest is a modification of Decision Tree that builds an ensemble of de-correlated trees based on bootstrap aggregation known as bagging; aggregation of prediction from individual trees can reduce the variance resulting in better predictive performance. AdaBoost on the other hand is an ensemble form of Decision Tree based on boosting that combines multiple weak classifiers in order to assign an appropriate weighting in the final voting to enhance the overall performance.

**Table 1:** Parameter specifications of different classification algorithms used in this study.

Algorithm	Descriptors
KNN	Number of neighbors (K_range) = 1–100, weights = 'uniform', algorithm = 'auto', leaf_size = 30, Power parameter for the Minkowski metric (p) = 2, metric = 'minkowski'
ANN	Max_iter = 1000 hidden_layer_sizes: 20 activation: ['tanh', 'relu'], solver: ['sgd', 'adam'], alpha: [0.0001, 0.05],
SVM	decision_function_shape = 'ovr', degree = 3, gamma = 'auto', kernel = 'rbf',
DT	criterion = 'gini', splitter = 'best', max_depth = 6, min_samples_split = 2, min_samples_leaf = 1,
RF	criterion = 'gini', max_depth = 6, min_impurity_decrease = 1e-07, min_samples_split = 2, n_estimators = 10,
AB	max_depth = 6 n_estimators = 600, learning_rate = 1.5, algorithm = ’SAMME’

**Figure 3:**
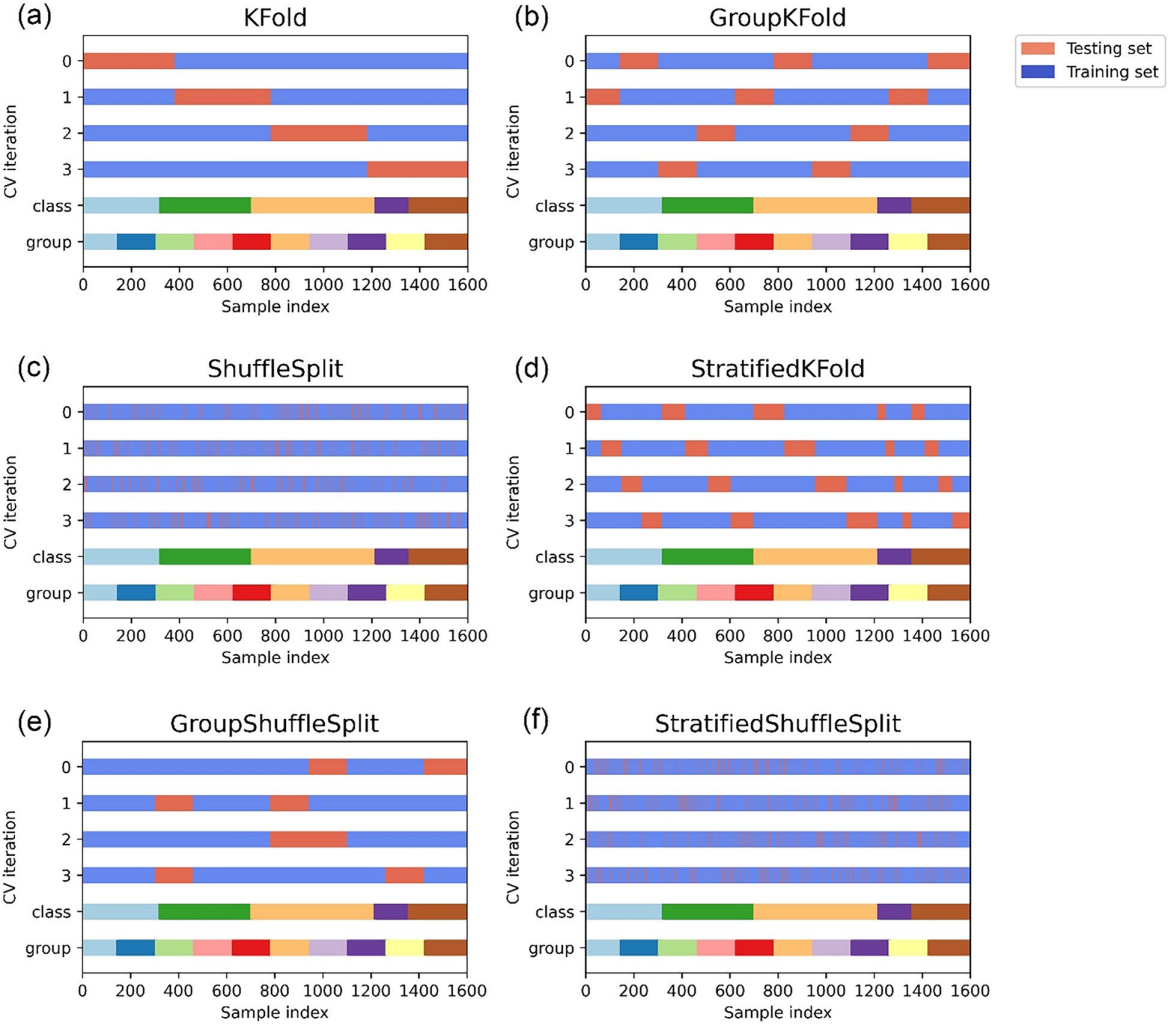
Data visualisation of different types of cross validation techniques performed in this study for generating random split of training and test sets. Data structure comprises of 1600 datapoints, 5 classes split unevenly across datapoints, and 10 groups split evenly across datapoints. The data is split into 4 folds where in on each split, the indices chosen for the training set (in blue) and the test set (in red) are visualised.

Different methods for splitting data were applied in conjunction with several cross-validation techniques to evaluate the accuracy of different algorithms using the cross-validation modules available in Scikit-learn library^34^. Figure 3 illustrates the different types of cross-validation techniques used for generating random splits of training and test datasets. In KFold cross-validator, the dataset is split into *k* consecutive folds and each fold is then used for validation while the *k*–1 remaining folds are used for training. Group KFold is a KFold variant with non-overlapping groups where the same group is not used in two different folds and are approximately balanced such that the number of distinct groups is the same in each fold. In shufflesplit, a random permutation is employed to yield indices to divide data for training and testing. However, contrary to other cross-validation strategies, random splits may not result in all the folds to be different. Another variation of K-Fold is Stratified KFold that returns stratified folds made by selecting samples from each class and ensuring the class strength is preserved. Further, in Group shufflesplit the group information is applied to obtain domain-specific stratifications in the form of integers for all the samples. However, the major difference compared to LeavePGroupsOut is that the splits are generated by all subsets of P unique groups, whereas in GroupShuffleSplit the splits are generated by the user based on random test splits, each with a user specified fraction of unique groups. Stratified ShuffleSplit is implemented by merging StratifiedKFold and ShuffleSplit that returns stratified randomized folds made by using appropriate percentage of samples in each class.

## Results and discussion

### Classification metrics

The classification ability of algorithms can be evaluated using multiple performance indicators, and often plays an important role in the design of the learning system. The selection of appropriate metrics is deemed to be as crucial as selecting the learning algorithm for a given classification problem. Accuracy is the most simple and easy to evaluate metric that can summarize a model’s performance. However, accuracy can often be misleading in imbalanced distributions because of the skewed nature of classes. Importantly, a measure of accuracy is unable to distinguish between the number of correctly classified examples of least represented or minority classes.

Accuracy is defined as:

4 $${\text{Accuracy}} = \frac{{\text{TP + TN}}}{{\text{TP + FN + TN + FP}}}$$

where TP and TN are the numbers of True Positives and True Negatives respectively; and FN and FP are the numbers of False Negatives and False Positives respectively.

**Figure 4:**
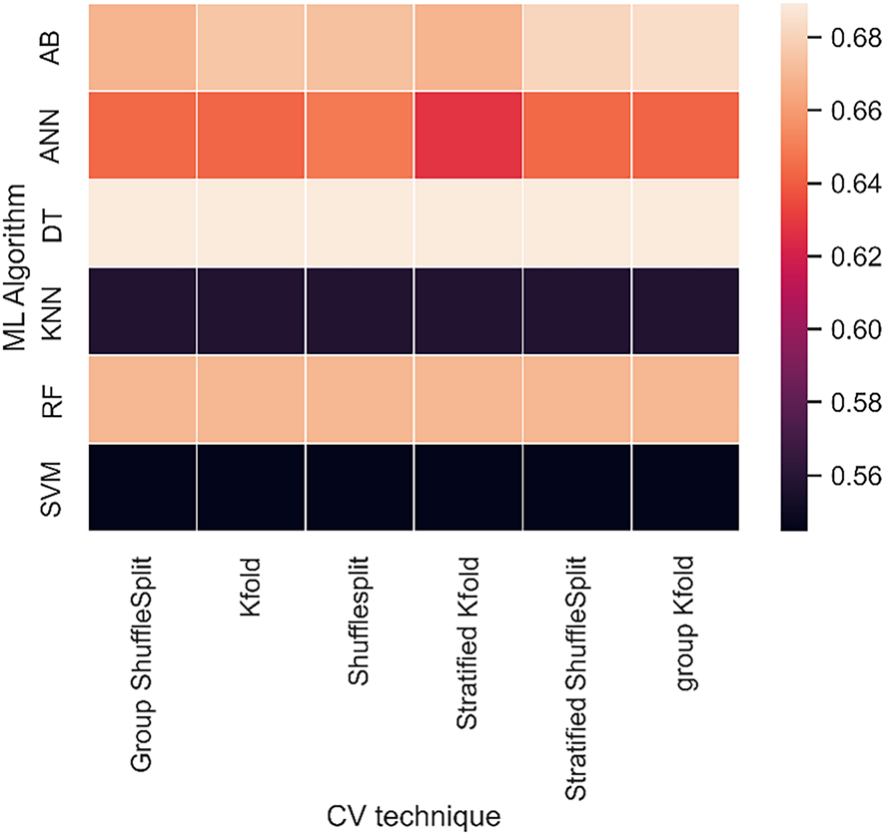
Heat map of cross validation accuracy (4 splits) using different machine learning algorithms. Among the classifiers used, Decision Trees (DT) followed by AdaBoost (AB) achieved the highest cross validation accuracy whereas K-Nearest Neighbours (KNN) and Support Vector Machines (SVM) reported the lowest metrics.

Figure 4 shows the heat map of cross validation accuracy (with 4 splits) using different algorithms. DT, RF and AB give the highest accuracy (in the range of 0.66–0.69) followed by ANN (accuracy range = 0.62–0.65). The cross-validation accuracy of KNN and SVM were observed to be the least with a value close to 0.55. Overall, the cross-validation accuracy has been found to be consistent regardless of the technique used to split the data.

Among classification metrics, a lot of emphasis is given to measures of precision and recall (sensitivity) as they are more effective in dealing with imbalanced distributions. However, it is difficult to achieve a trade-off between precision and recall parameters and the requirement is often dictated by the nature of the classification problem. Precision is the ratio between the true positives and all the positive cases observed, whereas Recall is the model’s ability to correctly identify true positives. Precision is more relevant when there is substantial interest to achieve high performance in identifying positive cases or there is higher margin for false negatives. Recall proves more reliable if the objective is to detect all the positives without fail or false positives are acceptable within certain margin. The Precision and Recall (true positive rate) terms can be expressed as:

5 $${\text{Precision = }}\frac{{{\text{TP}}}}{{\text{TP + FP}}}$$

6 $${\text{Recall (Sensitivity or TPR) = }}\frac{{{\text{TP}}}}{{\text{TP + FN}}}$$

In the context of threat detection, recall is more important than precision as false negatives can lead to severe consequences. However, there is an increasing desire to improve the efficiency of detector systems by reducing the rate of false positives. The F1-score is used when precision and recall are equally important (defined as the harmonic mean of Precision and Recall):

7 $${\text{F1 - score = 2}} \times \frac{{{\text{Precision }} \times {\text{ Recall}}}}{{\text{Precision + Recall}}}$$

Another classification metric called Specificity gives the measure of negative samples that are correctly classified. It is expressed as:

8 $${\text{Specificity (TNR) = }}\frac{{{\text{TN}}}}{{\text{TN + FP}}}$$

Classification metrics Precision, Recall, F1-score and Specificity for different classes are described in Table 2 (highest value of the metric is highlighted). DT is the winner with the precision metrics reportedly giving the highest values for both majority and minority classes. However, AB seems to be the most consistent with Recall metrics especially in minority classes. Furthermore, in the case of F1-score and Specificity, DT was found to give the highest metric values.

**Table 2:** Classification metrics Precision, Recall, F1-score and Specificity for different classes.

Metric	Algorithm	KNN	ANN	SVM	DT	RF	AB
	Class						
Precision	1	0.50	0.76	0.58	**0.78**	0.71	0.69
	2	0.49	0.53	**0.58**	0.54	0.54	0.53
	3	0.65	0.74	0.57	**0.78**	0.76	0.76
	4	0.40	0.50	0.58	**0.60**	0.49	0.53
	5	0.39	0.63	0.28	0.62	**0.66**	0.58
Recall	1	0.33	0.61	0.52	0.64	0.64	**0.65**
	2	0.61	**0.73**	0.27	0.68	0.58	0.54
	3	0.90	0.86	**0.98**	0.89	0.82	0.76
	4	0.32	0.37	0.39	0.39	0.47	**0.55**
	5	0.13	0.28	0.16	0.43	**0.57**	**0.57**
F1-score	1	0.40	0.68	0.55	**0.70**	0.67	0.67
	2	0.54	**0.61**	0.37	**0.61**	0.56	0.53
	3	0.76	0.80	0.72	**0.83**	0.79	0.76
	4	0.35	0.42	0.47	0.48	0.48	**0.53**
	5	0.20	0.39	0.21	0.50	**0.61**	0.58
Specificity	1	0.91	**0.95**	0.90	**0.95**	0.93	0.92
	2	0.80	0.79	**0.94**	0.82	0.84	0.87
	3	0.77	0.85	0.64	**0.88**	**0.88**	0.87
	4	0.96	0.97	**0.98**	**0.98**	0.96	0.95
	5	**0.97**	**0.97**	0.93	0.96	0.95	0.94

Classes are constituted as 1- Cobalt, 2- Caesium, 3- Iridium, 4- Uranium and 5- Thorium for the PGAA database used in this study.

Best metric values are indicated in bold.

Overall, tree-based algorithms were the clear winners with DT emerging as the best performer followed by RF and AB. Interestingly, tree-based algorithms have outperformed ANN taking into the account the performance metrics in both majority and minority classes, whereas SVM and KNN were found to be the weakest performers by a significant margin.

**Figure 5:**
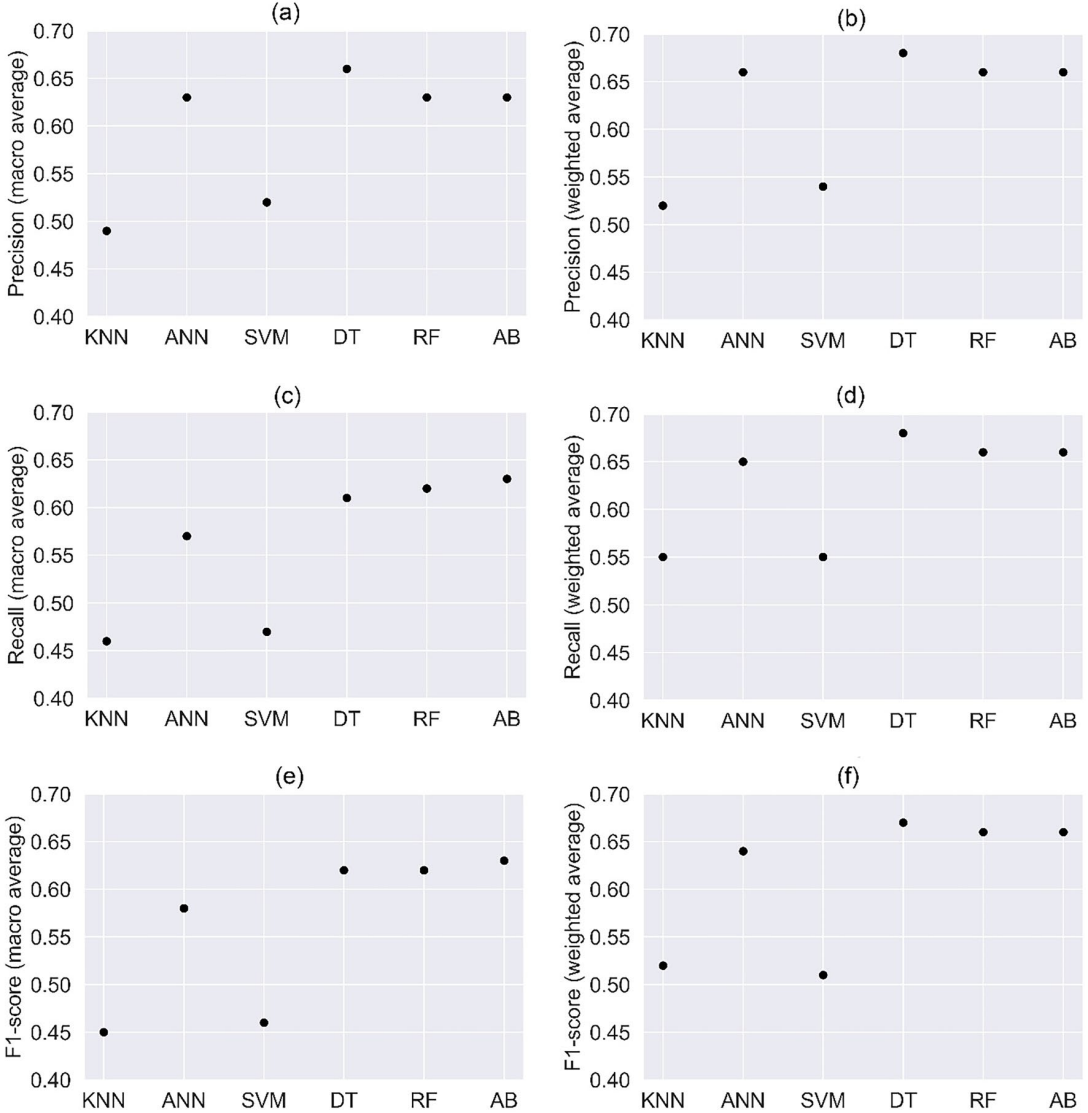
Macro and weighted average of precision, recall and F1-score evaluated using (**a**) K-Nearest Neighbours (**b**) Artificial Neural Networks (**c**) Support Vector Machines (**d**) Decision Trees (**e**) Random Forest (**f**) AdaBoost gradient algorithms. For a given metric *P*, Macro average is calculated using the aggregation process *P*_*1*_*class*_*1*_ + *P*_*2*_*class*_*2*_ + ….*P*_*n*_*class*_*n*_ results in greater penalisation when the model does not perform well in minority class. However, in weighted average the scores of independent class is added with a weight (*W*) using *P*_*1*_*class*_*1*_ × *W*_*1*_ + *P*_*2*_*class*_*2*_ × *W*_*2*_ + ….*P*_*n*_*class*_*n*_ × *W*_*n*_ favouring the majority class.

For better interpretability, the macro and weighted average of Precision, Recall, and F1 score are presented in Fig. 5. Macro average is calculated using the unweighted mean that can penalize the model if the performance in minority classes is poor. On the other hand, weighted average takes into account the number of true instances in each class to cope with class imbalance and consequently favours the majority class. DT has recorded the highest precision measures with macro and weighted averages, closely followed by ANN, RF and AB (see Fig. 5a,b). In the case of recall, AB has emerged as the best performer compared to the other tree-based algorithms with macro-average as a performance indicator. In the category of weighted average, DT is leading the performance chart followed by AB and RF (see Fig. 5c,d). In the case of F1-score, AB leads its counterparts based on the macro average and DT comes out as the top performer with weighted average (Fig. 5e,f). In comparison to tree-based algorithms, ANN is the only other algorithm that has shown similar level of performance, whereas KNN and SVM are seen to be ineffective for handling imbalanced classes. In summary, tree-based algorithms are found to be superior considering the precision, recall and F1-score indicators, with DT appearing more frequently on the leader board with weighted averages and AB with macro averages. Nevertheless, macro averages should be preferred over weighted averages in detection applications with skewed distributions as the former shows a true reflection of the performance in minority classes.

Further, we performed a Wilcoxon Sign-Ranked test on the classification metrics obtained from KNN, SVM, ANN, RF and AB (see Table 3) with the Null hypothesis that the difference between the pairs follows a symmetric distribution around zero. Samples were paired with metrics obtained from Decision Trees as it was the most consistent performer as evidenced in Table 2. The resulting *p*-value was insignificant (*p*-value > 0.05) as observed in DT-RF and DT-AB sample pairs. Hence the performance difference from the Tree-based methods can be considered as statistically insignificant. On the contrary, the performance metric was statistically better compared to the metrics obtained from KNN, SVM and ANN.

**Table 3:** Results from Wilcoxon Sign-Ranked test performed on classification metrics from K-Nearest Neighbour, Support Vector Machine, Artificial Neural Network, Random Forest and Ada Boost.

Paired samples	*T-*value	*p-*value
DT-KNN	3.0	9.53e−06
DT-SVM	18.5	0.0035
DT-ANN	19.0	0.0037
DT-RF	54.5	0.4847
DT-AB	75.5	0.2942

Samples are paired with metrics obtained from Decision Trees. The resulting *p*-value is insignificant (*p*-value > 0.05) in DT-RF and DT-AB sample pairs.

**Figure 6:**
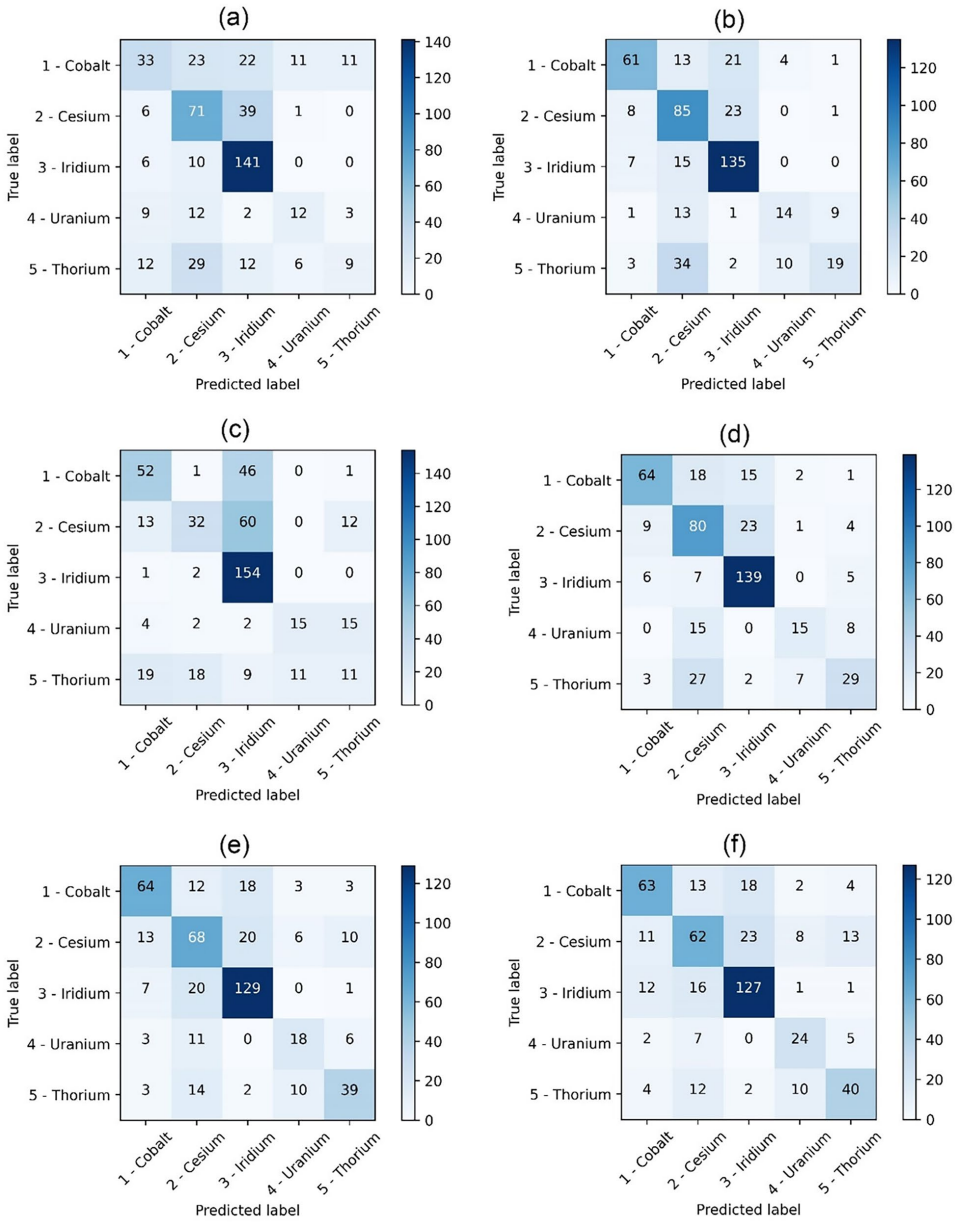
Confusion matrix obtained using: (**a**) K-Nearest Neighbours (**b**) Artificial Neural Networks (**c**) Support Vector Machines (**d**) Decision Trees (**e**) Random Forest (**f**) AdaBoost gradient algorithms. In a Confusion matrix (*C*_*ij*_) diagonal elements of the matrix denote the true positives, false positives (Type 1 error) can be evaluated by taking the sum of elements along the column (for class 1 the false positives can be expressed as the sum of *C*_*i1*_ elements (*i* = *1 to 5*)_._ The total number of false negatives (Type 2 error) in class 1 can be calculated by taking the sum of row elements i.e. the sum of *C*_*1j*_, (*j* = *1 to 5*).

A Confusion Matrix provides a graphical representation of the classification results by comparing the true labels and the predicted labels. The matrix (*C*_*ij*_) takes the form of an *N* × *N* square matrix where *N* is the number of target classes such that true positives are represented as the diagonal elements of the matrix. The total number of false positives (Type 1 error) in a class can be evaluated by taking the sum of elements along the column excluding the diagonal elements of the square matrix. For instance, for class 1 the false positives can be expressed as the sum of *C*_*i1*_ elements (*i* = 1 to 5)_._ Similarly, the total number of false negatives (Type 2 error) in a class can be calculated by taking the sum of row elements: *i.e.*, for class 1, the sum of *C*_*1j*_, (*j* = 1–5). Figure 6 shows the Confusion matrix obtained using different machine learning algorithms. The Type 1 (FP) and Type 2 (FN) errors can be directly calculated from the Confusion matrix. Table 4 presents a comparison of Type 1 and Type 2 errors for majority and minority classes for different algorithms. The least Type 1 error of 40 in the majority class is reported with DT and RF algorithms out of 157 classifications where highest number of false positives is observed in SVM. In minority class, DT has superior performance with just 10 cases of false positives among 38 observations. However, when it comes to Type 2 error, SVM reports the best performance of just 3 out of 271 observations in the majority class. This clearly shows a systematic bias in SVM with very high recall value in the majority class whilst demonstrating poor performance in other classes. The frequency of false negatives (Type 2 error) in minority class is lowest with AB and validates the high recall reported in the minority class. Overall, tree-based algorithms are the most consistent with DT demonstrating slightly better performance in Type 1 error and AB in Type 2 error.

**Table 4:** Comparison of Type 1 and Type error for majority and minority classes for different algorithms. Class 3 (Iridium) represents the majority class and Class 4 (Uranium) stands for the minority class.

Algorithm	Type 1 error (False positives)		Type 2 error (False negatives)	
	Majority class	Minority class	Majority class	Minority class
KNN	75	18	16	26
ANN	47	14	22	24
SVM	117	11	**3**	23
DT	**40**	**10**	18	23
RF	**40**	19	28	20
AB	43	21	30	**14**

Best metric values are indicated in bold.

### ROC curves and AUC score

**Figure 7:**
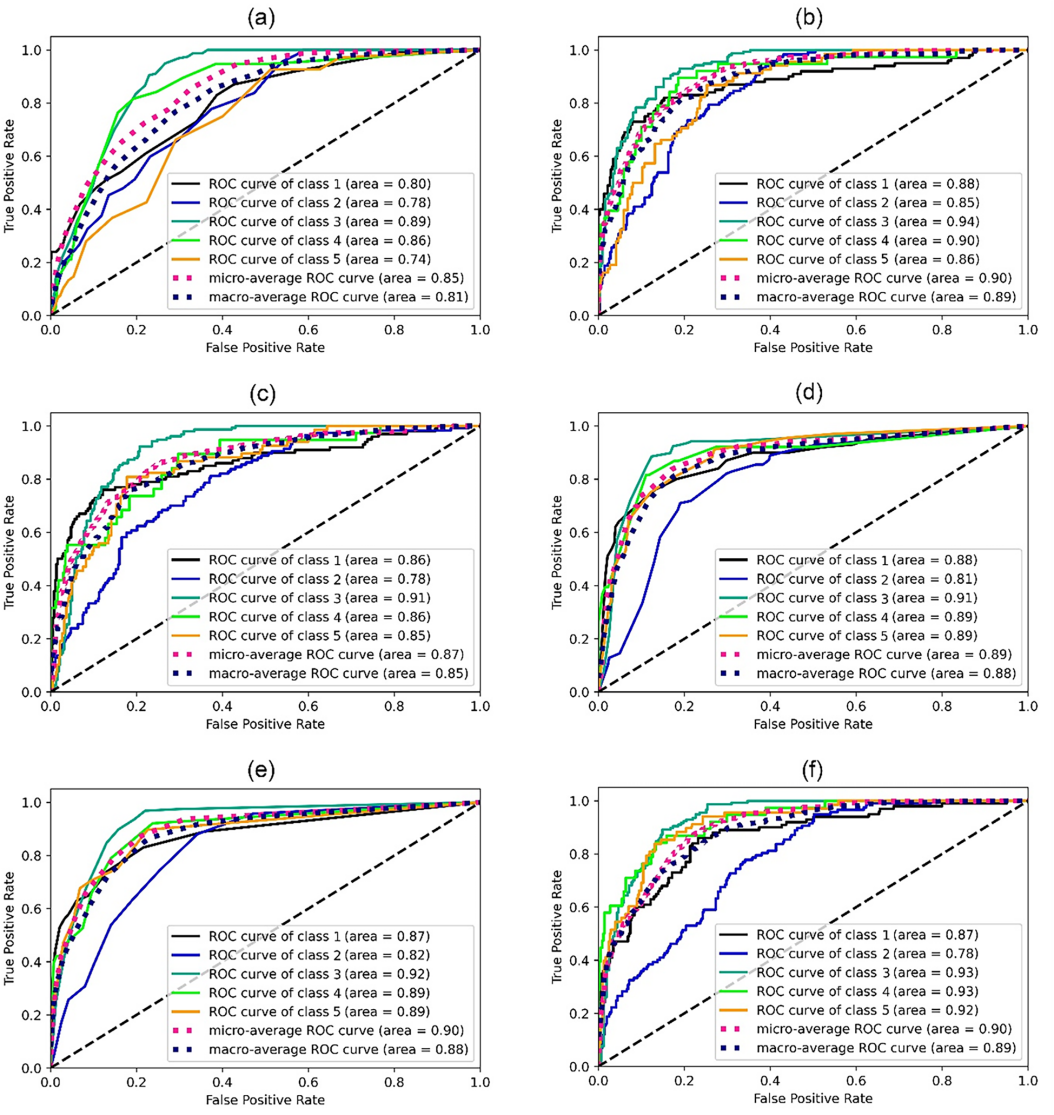
Receiver operating characteristic (ROC) curves using One-vs-Rest scheme: (**a**) K-Nearest Neighbours (**b**) Artificial Neural Networks (**c**) Support Vector Machines (**d**) Decision Trees (**e**) Random Forest (**f**) AdaBoost gradient algorithms. In the ROC curve, micro-average was calculated by aggregating the contributions of all classes whereas macro-average gives equal weight to the classification of each class.

The Receiver Operating Characteristic (ROC) is a trend curve used to quantify the multi-class classification performance of various threshold settings^34,36^. ROC represents a probability curve that can give some insight on how capable a model is to distinguish different classes. ROC is a plot of the True Positive Rate (TPR) on the Y-axis and the False Positive Rate (FPR) on the X-axis. The steepness associated with ROC curves signify to what extent the model is capable of maximising the TPR while minimizing the FPR. Area Under the Curve (AUC) is another performance measure used to demonstrate the model’s ability to separate different classes. The higher the value of the AUC, the better its ability to strike a balance between sensitivity and specificity. The AUC can be very effective whilst dealing with imbalanced distributions, and can avoid overfitting to a single class. In order to generate ROC curves, averaged set of TPR against fixed FPR was obtained after binarizing the output using One-vs-Rest scheme that compares each class against all the others (assumed as one). The predicted labels are provided in an array with values ranging from 1 to 5 (number of classes), and the scores correspond to the probability estimates that a sample belongs to a particular class. Figure 7 shows the Receiver Operating Characteristics (ROC) curves using different machine learning algorithms using One-vs-Rest scheme. The highest micro and macro average was obtained using ANN and AB algorithms. However, AB has the competitive edge as it gives the highest class-wise metric for the minority class. Other tree-based algorithms DT and RF also performed appreciably well and closely match the results of AB and ANN. Table 5 shows the AUC score obtained by using different machine learning algorithms. In One-vs-Rest (OvR), the AUC is evaluated based on each class against the rest and is sensitive to class imbalance. On the contrary, the multi-class One-vs-One (OvO) scheme compares every unique pairwise combination of classes and is not sensitive to class imbalance. The AUC was calculated using the OvR and OvO schemes for computing both macro average and prevalence-weighted average. ANN reportedly indicate the highest macro and weighted average in OvR whereas AB emerged as the most superior in the OvO scheme clearly showing the ability to deal with skewed classes.

**Table 5:** Area under the curve (AUC) score obtained from different ML algorithms used in this study.

AUC score	One-vs-Rest		One-vs-One	
	macro	weighted by prevalence	macro	weighted by prevalence
KNN	0.8129	0.8200	0.7865	0.8067
ANN	**0.8850**	**0.8898**	0.8610	0.8789
SVM	0.8526	0.8554	0.8340	0.8478
DT	0.8760	0.8747	0.8648	0.8648
RF	0.8801	0.8810	0.8734	0.8794
AB	0.8824	0.8774	**0.8769**	**0.8822**

Best metric values are indicated in bold.

### Extended metrics

The classification performance is evaluated based on some extended metrics that are specific to imbalanced distributions. One such metric is the Geometric Mean Score (GMS) defined as the *n*-th root of the product of sensitivity of each class. This measure effectively maximizes the accuracy of each class while obtaining good balance. Although GMS can reduce the inhibitory effects of skewed classes, it cannot separate the contribution of each class to the overall performance or reduce the effect of the dominant class. Another metric called the Index Balanced Accuracy (IBA) can be defined for any performance metric. The advantage of using IBA is that it takes into account the effect of the dominant class by utilizing a scoring function. Here we evaluate the IBA of GMS expressed as:

9 $${\text{GMSIBA}} = (1 + \alpha {\kern 1pt} .{\kern 1pt} \,{\text{Dominance}}){\kern 1pt} {\kern 1pt} .{\kern 1pt} {\kern 1pt} {\text{Gmean}}^{2}$$

where Dominance is an index to measure to signify the dominance of the majority class and *α* is a term that defines the weighting factor. In the present work we used the default value of *α* = 0.1.

10 $${\text{Dominance = TPR - FPR}}$$

**Figure 8:**
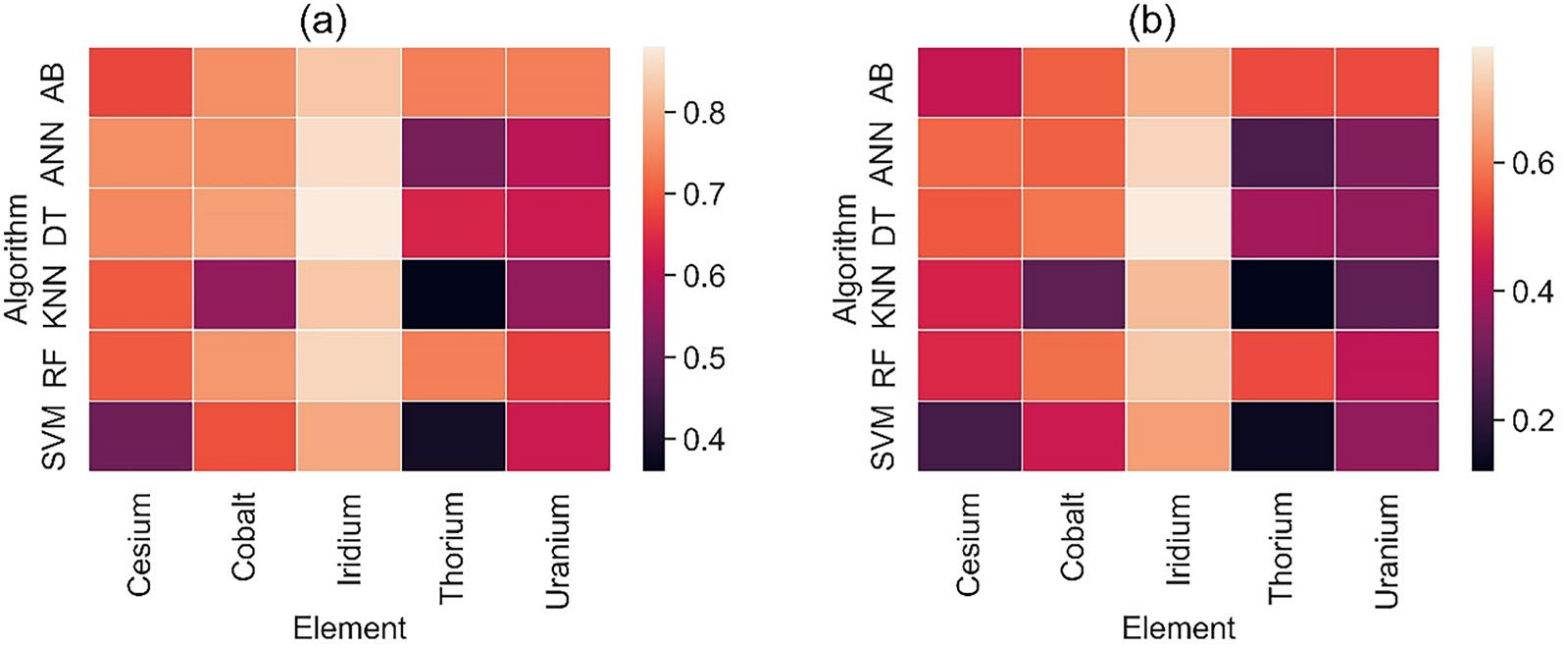
Heat map showing the variation of (**a**) Geometric Mean Score (GMS) and (**b**) Index Balanced Accuracy of geometric mean score (GMS_IBA_) for different algorithms. With both metrics, Decision Trees showed the highest score in the majority classes whereas AdaBoost was the lead performer in the minority classes (Uranium and Thorium).

Figure 8 shows the Heat map showing the class-wise distribution of the Geometric Mean Score (GMS) and Index Balanced Accuracy of GMS. Both metrics follow similar patterns in the class-wise distributions. DT is the best performer in the majority class, followed by ANN and then RF and AB. However, AB performs better than any other algorithms with the metrics associated with the minority class by a significant margin. We also present the mean values of geometric mean score (GMS) and index-balanced accuracy of GMS (GMS_IBA_) evaluated using different ML algorithms in Table 6. Here, DT is the winner closely followed by RF and AB. Overall, Tree-based algorithms lead the chart of performance metrics among the classification algorithms. Interestingly, the superior performance of AB in the minority class is not reflected in the mean metrics of GMS and GMS_IBA_. This emphasises why it is important to consider the class-wise distributions rather than evaluating a global metric in the case of skewed distributions.

**Table 6:** Mean values of geometric mean score (GMS) and index balanced accuracy (IBA) evaluated using different ML algorithms.

Algorithm	Metrics	
	GMS (mean)	GMS_IBA_ (mean)
KNN	0.65	0.44
ANN	0.75	0.56
SVM	0.63	0.41
DT	**0.77**	**0.59**
RF	0.77	0.58
AB	0.76	0.57

Best metric values are indicated in bold.

Overall, the classification metrics imply that the tree-based algorithms and ANN have performed exceedingly well, whilst SVM and KNN displayed poor performance and were highly inconsistent. Both KNN and SVM have been particularly susceptible to class imbalance and are biased towards the majority instances of the training space. However, it is not a straightforward task to select a winner despite the majority of the classification metrics suggesting DT as the best-performing algorithm. This is because in the context of threat detection of illicit materials, the algorithm must be able to detect all the positive cases (flagged as potential threats) from a very large sample space dominated by non-threat classes. Hence, recall is more important than precision and the ability to identify false negatives in the minority classes plays a crucial role in determining the efficacy of an algorithm. This has been a major weakness associated with DT despite the fact that it has been consistent and leads the performance metric in multiple categories. Hence, the performance of DT may be greatly influenced by the contribution of the dominant classes. In contrast, AB has been more consistent and much more superior with Recall metric and performance in the minority class. Moreover, AB was found to be leading in the ROC-AUC metric and reportedly the top performer in the GMS within the minority class. Furthermore, the classification metrics used in this study have been helpful in comparing different ML algorithms. However, the metrics should be carefully chosen based on the application domain and more weighting should be given to the metrics to address the precision-recall trade-off and the class-wise distribution of the spectral data. Furthermore, as a recommendation for selecting the best algorithm in skewed distributions, the class-wise sensitivity of the metrics should be considered rather than evaluating a global metric that may favour the majority classes in the spectral data.

In practice, the consequences of misclassification can have serious security and cost implications depending on the impact on the type of application within the nuclear context. Importantly, false alarm (false positive) in nuclear security may be seen as the case which has less severe consequences than a missed classification (false negative). For instance, in a hidden source scenario (e.g., cargo scanning) if the caesium is misclassified as uranium, then there is a need for further detailed search that involves manual inspection and results in higher costs. On the other hand, the consequences may be less severe if caesium is misclassified as cobalt as it is not a special nuclear material. However, the misclassification of caesium with cobalt may raise an alarm in the facility as it indicates the illicit movement of cobalt, which may have been stolen from the premises undertaking radiological therapies and can lead to additional financial liabilities to validate the finding.

## Conclusions

In this study, prompt-gamma activation analysis (PGAA) spectral data are used to examine the classification performance of different Machine Learning algorithms for threat detection of radiological materials. Several classification metrics were used undertake to perform robust comparative studies of different Machine Learning algorithms. The main conclusions of this study can be summarized as follows,

- Tree-based classifiers are found to be superior considering the precision, recall, F1- score, Receiver Operating Characteristics, Area under the Curve and Geometric Mean Score indicators to analyse PGAA spectral data with imbalanced class distributions. Tree-based algorithms have consistently outperformed Support Vector Machines and K-Nearest Neighbours whilst in comparison, Artificial Neural Networks is the only other algorithm that has shown similar level of performance. In general, K-Nearest Neighbours and Support Vector Machines are found to be ineffective for handling PGAA spectral data with imbalanced class distributions.
- Decision Tree reported the highest values with majority of the classification metrics but this outcome was greatly influenced by the contribution of the dominant classes and demonstrated a bias towards high precision. However, based on the results presented, AdaBoost is the preferred classifier to analyse PGAA data due to the high recall and least number of false negatives in the minority classes.
- The classification metrics used in this study have been helpful in comparing the effectiveness of different Machine Learning algorithms for classifying PGAA data. However, in detection applications, the metrics should be carefully chosen in order to address the precision-recall trade-off and the class-wise distribution of the spectral data. Specifically, it is recommended that the algorithm for application in skewed distributions should be selected based on class-wise sensitivity of the metrics rather than evaluating a global metric that is deemed less reliable as it favours the majority classes.

## Supplementary Information

Supplementary Information.

## Data Availability

The datasets analysed during the current study are available in the PGAA prompt gamma data repository, using the link: https://www-nds.iaea.org/pgaa/databases.htm.

## Abbreviations

PGAA: Prompt gamma ray neutron activation analysis
ML: Machine learning
KNN: K-nearest neighbours
ANN: Artificial neural network
SVM: Support vector machines
DT: Decision trees
RF: Random forest
AB: AdaBoost
TP: True positive
TN: True negative
FP: False positive
FN: False negative
FPR: False positive rate
TNR: True negative rate
ROC: Receiver operating characteristics
AUC: Area under the curve
GMS: Geometric mean score
IBA: Index balanced accuracy

## References

[1] The Royal Society. Detecting nuclear and radiological materials. RS policy document 07/08 (2008).

[2] IAEA. Nuclear security systems and measures for major public events. *IAEA Nucl. Secur. Ser.* (2012).

[3] IAEA. Database of prompt gamma rays from slow neutron capture for elemental analysis. 251 (2007).

[4] Perry DL (2002). Neutron-induced prompt gamma activation analysis (PGAA) of metals and non-metals in ocean floor geothermal vent-generated samples. J. Anal. At. Spectrom..

[5] Belgya T (2012). Prompt gamma activation analysis at the budapest research reactor. Phys. Procedia.

[6] Im HJ, Song K (2009). Applications of prompt gamma ray neutron activation analysis: Detection of illicit materials. Appl. Spectrosc. Rev..

[7] Yoshida E, Shizuma K, Endo S, Oka T (2002). Application of neural networks for the analysis of gamma-ray spectra measured with a Ge spectrometer. Nucl. Instrum. Method Phys. Res. Sect. A Accel. Spectrometer Detect. Assoc. Equip..

[8] Shue SL, Faw RE, Shultis JK (1998). Thermal-neutron intensities in soils irradiated by fast neutrons from point sources. Chem. Geol..

[9] Alamaniotis M, Heifetz A, Raptis AC, Tsoukalas LH (2013). Fuzzy-logic radioisotope identifier for gamma spectroscopy in source search. IEEE Trans. Nucl. Sci..

[10] Alamaniotis M, Jevremovic T (2015). Hybrid fuzzy-genetic approach integrating peak identification and spectrum fitting for complex gamma-ray spectra analysis. IEEE Trans. Nucl. Sci..

[11] Fatah AH, Ahmed AH (2011). Analysis of gamma-ray spectra using Levenberg-Marquardt method. World Acad. Sci. Eng. Technol..

[12] Varley A, Tyler A, Smith L, Dale P, Davies M (2016). Mapping the spatial distribution and activity of ^226^Ra at legacy sites through Machine Learning interpretation of gamma-ray spectrometry data. Sci. Total Environ..

[13] Sullivan CJ, Stinnett J (2015). Nuclear instruments and methods in physics research a validation of a Bayesian-based isotope identification algorithm. Nucl. Inst. Methods Phys. Res. A.

[14] Kamuda M, Stinnett J, Sullivan CJ (2017). Automated isotope identification algorithm using artificial neural networks. IEEE Trans. Nucl. Sci..

[15] Kamuda M, Sullivan CJ (2019). An automated isotope identification and quantification algorithm for isotope mixtures in low-resolution gamma-ray spectra. Radiat. Phys. Chem..

[16] Im HJ, Song BC, Park YJ, Song K (2009). Classification of materials for explosives from prompt gamma spectra by using principal component analysis. Appl. Radiat. Isot..

[17] Nunes WV, Da Silva AX, Crispim VR, Schirru R (2002). Explosives detection using prompt-gamma neutron activation and neural networks. Appl. Radiat. Isot..

[18] Hossny K, Hossny AH, Magdi S, Soliman AY, Hossny M (2020). Detecting shielded explosives by coupling prompt gamma neutron activation analysis and deep neural networks. Sci. Rep..

[19] Shahabinejad H, Vosoughi N, Saheli F (2020). Matrix effects corrections in prompt gamma-ray spectra of a PGNAA online analyzer system using artificial neural network. Prog. Nucl. Energy.

[20] Cheng, K.Y., Shayan, H., Krycki, K., Hegermann, L.M. Prompt Gamma ray neutron activation analysis (PGNAA) Metal spectral classification using deep learning method. arXiv:2208.13909v1 [cs.LG] (2022).

[21] Zolfaghari M, Masoudi SF, Rahmani F, Fathi A (2022). Thermal neutron beam optimisation for PGNAA applications using Q-learning algorithm and neural network. Sci. Rep..

[22] Kamuda M, Zhao J, Huff K (2020). A comparison of machine learning methods for automated gamma-ray spectroscopy. Nucl. Instrum. Method Phys. Res. Sect. A Accel. Spectrometer. Detect Assoc. Equip..

[23] Spooner A (2020). A comparison of machine learning methods for survival analysis of high-dimensional clinical data for dementia prediction. Sci. Rep..

[24] Uddin S, Khan A, Hossain E, Moni MA (2019). Comparing different supervised machine learning algorithms for disease prediction. BMC Med. Inform. Decis. Mak..

[25] Analysis J, Quantitative OF (2019). Comparison of different machine learning algorithms for lithofacies comparison of different machine learning algorithms for lithofacies classification from well logs. Boll. Geofis. Teor. Appl..

[26] Kubat, M. and Matwin, S. Addressing the curse of imbalanced training sets: one-sided selection, *ICML* (1997).

[27] Barandela R, Sánchez JS, Garcıa V, Rangel E (2003). Strategies for learning in class imbalance problems. Pattern Recogn..

[28] García V, Sánchez JS, Mollineda RA (2012). On the effectiveness of preprocessing methods when dealing with different levels of class imbalance. Knowl. Based Syst..

[29] Branco, P., Torgo, L. & Ribeiro, R. A survey of predictive modelling under imbalanced distributions. *arXiv*:**1505**, 1–48 (2015).

[30] Nega A, Giacobini M, Michalak K (2021). A review of methods for imbalanced multi-label classification. Pattern Recogn..

[31] Molnar GL, Revay Z, Belgya T, Firestone RB (2000). The new prompt gamma-ray catalogue for PGAA. Appl. Radiat. Isot..

[32] Krawczyk B (2016). Learning from imbalanced data: Open challenges and future directions. Prog. Artif. Intell..

[33] Albon, C. Machine Learning with Python Cookbook: Practical Solutions from Preprocessing to Deep Learning (1st. ed.). O'Reilly Media, Inc. (2018).

[34] Pedregosa F (2011). Scikit-learn: Machine learning in python. J. Mach. Learn. Res..

[35] Bishop CM (2006). Pattern Recognition and Machine Learning (Information Science and Statistics).

[36] Fawcett T (2006). An introduction to ROC analysis. Pattern Recognit. Lett..

